# Use of Stress Signals of Their Attached Bacteria to Monitor Sympagic Algae Preservation in Canadian Arctic Sediments

**DOI:** 10.3390/microorganisms9122626

**Published:** 2021-12-20

**Authors:** Rémi Amiraux, Bonin Patricia, Burot Christopher, Rontani Jean-François

**Affiliations:** 1 CNRS/INSU/IRD, Mediterranean Institute of Oceanography (MIO), UM 110, Aix-Marseille University, Université de Toulon, 13288 Marseille, France; remi.amiraux@takuvik.ulaval.ca (R.A.); patricia.bonin@mio.osupytheas.fr (B.P.); christopher.burot@mio.osupytheas.fr (B.C.); 2 UMR 6539 Laboratoire des Sciences de l’Environnement Marin, (CNRS, UBO, IRD, Ifremer) Institut, Universitaire Européen de la Mer (IUEM), 29280 Plouzané, France; 3 Takuvik Joint International Laboratory, Département de Biologie, Laval University (Canada)—CNRS, Université Laval, Québec, QC G1V 0A6, Canada

**Keywords:** sympagic algae, Arctic, bacterial stress, lipid tracers, sediments, preservation

## Abstract

Based on the strong aggregation of sympagic (ice-associated) algae and the high mortality or inactivity of bacteria attached to them, it was previously hypothesized that sympagic algae should be significant contributors to the export of carbon to Arctic sediments. In the present work, the lipid content of 30 sediment samples collected in the Canadian Arctic was investigated to test this hypothesis. The detection of high proportions of *trans* vaccenic fatty acid (resulting from *cis-trans* isomerase (CTI) activity of bacteria under hypersaline conditions) and 10*S*-hydroxyhexadec-8(*trans*)-enoic acid (resulting from 10*S*-DOX bacterial detoxification activity in the presence of deleterious free palmitoleic acid) confirmed: (i) the strong contribution of sympagic material to some Arctic sediments, and (ii) the impaired physiological status of its associated bacterial communities. Unlike terrestrial material, sympagic algae that had escaped zooplanktonic grazing appeared relatively preserved from biotic degradation in Arctic sediments. The expected reduction in sea ice cover resulting from global warming should cause a shift in the relative contributions of ice-associated vs. pelagic algae to the seafloor, and thus to a strong modification of the carbon cycle.

## 1. Introduction

In the Canadian Arctic, primary production is supported by sympagic (ice-associated) algae dominated by pennate diatoms and more specifically by the Naviculaceae [1] or by the centric diatom *Melosira arctica* [2] during the ice-covered period, and then by phytoplankton in open waters [3,4]. The contribution of sympagic algae to total primary production varies widely depending on the season and the region [5,6]. Because of global warming (causing a decrease in sea ice extent and duration), we are currently witnessing a reduced primary production of the sympagic algae and an increase in that of pelagic phytoplankton. Sympagic algae are assumed to be a main source of organic matter reaching the seafloor [2,7,8,9]. It is therefore feared that in the future, the biological pump would have a positive feedback effect on global warming. The high contribution of sympagic algae to Arctic sediments was previously attributed to: (i) their strong aggregation resulting from the high concentrations of extracellular polymeric substances (EPSs) produced by these organisms in the ice, which protects biogenic silica of diatom frustules from dissolution [10] and induces quick settling in the water column (100–500 m d^−1^; [11]); (ii) the fact that aggregated microalgae are not favored prey particles for grazers [12]; and (iii) the poor physiological status of their associated bacterial communities (inducing weak mineralization [7] and biogenic silica dissolution [13] in the water column and surface sediments). These bacterial communities are damaged by: (i) intense osmotic stress induced by changes in salinity in brine channels during the early stage of ice melting [7], and (ii) the production of bactericidal free fatty acids (FFAs) by sympagic algae latter in the season under the effect of light stress [14].

It is well-known that bacteria colonize nearly all types of particulate organic matter (POM) (e.g., phytodetritus, zooplanktonic fecal pellets, aggregated sympagic algae, and EPS particles [15]). Attachment of bacteria to particles may be favored by the presence of EPSs, which can act as biological glue [16,17]. In sea ice, more than 50% of the bacterial community were found to be associated with particles [18], while the contribution of attached bacteria to the total bacterial production was found to be highly variable in POM (ranging from 0 to 98% in the Beaufort Sea [19,20]). Particle attachment is very important for the sinking export of bacteria, since the sinking velocities of attached bacteria are significantly higher than those of free-living bacteria [21]. This partly explains the overwhelming proportion of bacteria attached to particles in marine sediments [22].

To survive under hypersaline conditions, bacteria have developed various strategies, such as (i) implementation of active Na^+^ and K^+^ ion transport systems [23]; (ii) accumulation of osmocompatible compounds such as glycine betaine or proline [24]; or (iii) production of EPSs, which can act as a diffusion barrier [25]. Another major adaptive response of bacteria of the *Pseudomonas* and *Vibrio* genera is to maintain their membrane fluidity by conversion of *cis* to *trans* unsaturated fatty acids through the activity of *cis-trans* isomerases (CTIs) [26,27]. Guckert et al. [28] have proposed a *trans/cis* ratio >0.1 as an indicator of bacterial stress. A relatively strong CTI activity was previously observed in sea ice and sinking particles collected at the beginning of the melting period in western Baffin Bay (*trans/cis* ratio and *trans*-vaccenic flux ranging at 25 m from 0.12 to 0.50 g:g and 0 to 4.0 µg d^−1^ m^−2^, respectively) [7,8] and was attributed to the flush of bacteria associated with sympagic algae from internal hypersaline ice brines [14]. The relative stability of *trans/cis* ratios with depth suggested that bacterial communities associated with sinking sympagic algae were non-growing and thus inactive. Indeed, in the absence of osmotic stress (as is the case in the water column), the *trans/cis* ratio of bacteria should decrease to a base level [29], requiring de novo synthesis of *cis* fatty acids [30] and thus bacterial growth. The similarity of these ratios observed in the water column and in sediments of western Baffin Bay (mean value 0.22 and 0.23 g:g, respectively [7]) is also indicative of a weak colonization of sinking particles and sediments by unstressed pelagic and benthic bacteria.

FFAs damage bacterial cellular membranes [31,32]. These compounds can also inhibit some enzyme activity, disrupt electron transport chains, and uncouple oxidative phosphorylation [33]. Bacteria attached to sympagic diatoms are particularly sensitive to the antibacterial activity of free palmitoleic acid produced by these algae under increasing light intensity [34]. To detoxify this acid, some bacteria of the *Pseudomonas, Pseudoalteromonas,* and *Shewanella* genera [35,36] present in Arctic sea ice [37] use a 10-dioxygenase (10*S*-DOX) able to convert palmitoleic acid to 10*S*-hydroperoxyhexadec-8(*trans*)-enoic acid. The flux of this enzymatic oxidation product previously measured in western Baffin Bay at 25 m ranged from 0 to 93 µg m^−2^ d^−1^ [7]. Despite this detoxification strategy, most of the bacteria associated with sinking sympagic algae (up to 70%) at the end of ice melting (when osmotic stress ceased) have disrupted membranes and may thus be considered dead [14]. However, importantly, 10*S*-DOX may also be utilized by bacteria to detoxify FFAs released by wounded pelagic diatoms in the presence of copepods [38].

Given that: (i) only bacteria attached to particles (such as aggregated phytodetritus) possess sufficient sinking rates to reach the seafloor and (ii) in the Arctic, sea ice is the largest source of salinity stress (owing to its extent and brine contents), previous observation of high proportions of *trans* monounsaturated fatty acids in some Arctic sediment samples [7,8,39] was attributed to the presence of sea ice biota in this material.

**Figure 1:**
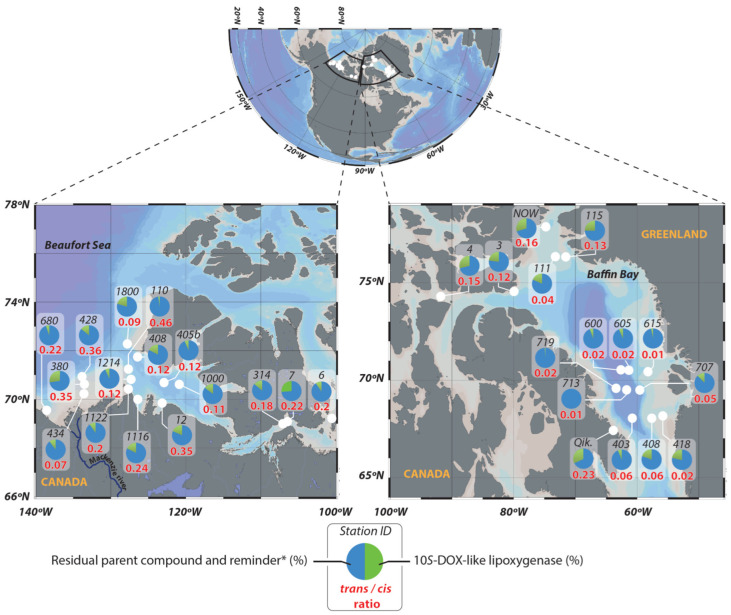
Map of the station investigated in the Beaufort Sea and Baffin Bay. The pie charts show the percentage of residual parent compounds (palmitoleic acid) degraded by the 10*S*-DOX. In red, the *trans/cis* vaccenic acid ratio. Qik stands for Qikiqtarjuaq. (*) abiotic oxidationproducts.

To confirm this expected strong contribution of sympagic material to the seafloor, we examined the lipid content of 30 sediment samples collected in the Canadian Arctic (Figure 1). Despite the recent development of several indices based on the relative proportions of highly branched isoprenoids (HBIs; IP_25_ and tri-unsaturated HBIs) to estimate relative proportions of sympagic vs. pelagic production [40,41,42,43], some uncertainties remain about the nature of POM reaching the seafloor. In particular, processes affecting the sinking organic matter and its lipid content through the water column down to the seafloor (such as degradation and remineralization) need further investigation. The aim of this work was thus to use new indicators of the osmotic and chemical stress status of bacteria associated with sympagic material (*trans*-vaccenic acid and 10*S*-hydroxyhexadec-8(*trans*)-enoic acid, respectively) to estimate: (i) the contribution of the sympagic material to the investigated sediments and (ii) the status of the bacteria associated with it. Ratios of stanols (widespread bacterial biohydrogenation products of sterols [44,45]) to their parent sterols were also used to confirm the impaired ability of stressed bacteria to degrade sympagic material.

## 2. Materials and Methods

### 2.1. Sediment Sampling

Two contrasting regions were selected, namely the Canadian Beaufort Shelf and Baffin Bay. The Beaufort Shelf is a perennially stratified interior shelf influenced by Pacific-derived waters supplied via the Beaufort Gyre and the Alaskan coastal current [46]. Sediments of this seasonally ice-covered shelf are strongly influenced by the Mackenzie River [47], the largest river draining into the Arctic in terms of sediment and POM [48]. The Mackenzie shelf functions as a vast estuary receiving inputs of both terrestrial and marine sources of organic matter [49]. By contrast, Baffin Bay is characterized by a strong spatial sea ice variability resulting from the inflow of the West Greenland Current (WGC) composed of the relatively warm Atlantic Irminger Current (IC), which restricts sea ice extent in its eastern part [50]. The sedimentation rates appeared to be relatively similar in the two zones investigated ranging from 0.04 to 0.20 cm yr^−1^ in the Beaufort Shelf [51,52] and from 0.06 to 0.11 cm yr^−1^ in Baffin Bay [52]. Depths of the different sampling stations (ranging from 7 to 2017 m) are given in Supplementary Table S2.

Sediment samples were collected from a broad range of locations (Figure 1) within the Canadian Arctic using an USNEL box corer (50 × 50 × 40 cm^3^) on board the CCGS Amundsen in 2005 (ArcticNet survey), 2008 (IPY-CFL), 2009 (Malina campaign), and 2015 and 2016 (GreenEdge campaigns). Although a significant decrease in ice concentration under the effect of global warming between 2005 and 2016 was logically to be expected [53], vertical particulate organic carbon (POC) exports recorded in the Mackenzie Shelf from 1987 to 2006 (ranging from 1.6 to 1.8 g C m^−2^ yr^−1^ [54,55,56]) seemed relatively unaffected. Moreover, similar values were measured in the Amundsen Gulf (ranging from 2.4 and 6.8 g C m^−2^ yr^−1^ [56,57]) and in Baffin Bay (ranging from 1.1 to 6.7 g C m^−2^ yr^−1^ [56,58]) justifying a comparative study of the different sediment samples investigated. From each box core, one sample of ca. 50 cm^2^ was collected from intact sediment surface (0–1 cm) and frozen immediately at −80 °C for later analysis.

### 2.2. Lipid Analysis

Samples were reduced with excess NaBH_4_ after addition of MeOH (25 mL, 30 min) to reduce labile hydroperoxides to alcohols, which are more amenable to analysis using gas chromatography-mass spectrometry (GC–MS). Water (25 mL) and KOH (2.8 g) were then added and the resulting mixture saponified (to break complex lipids down into their constituent fatty acids) by refluxing (2 h). After cooling, the mixture was acidified (HCl, 2 N) to pH 1 and extracted with dichloromethane (DCM; 3 × 20 mL). The combined DCM extracts were dried over anhydrous Na_2_SO_4_, filtered and concentrated by rotary evaporation at 40 °C to give total lipid extracts (TLEs). Aliquots of TLEs were either silylated and analyzed by gas chromatography-electron impact quadrupole time-of-flight mass spectrometry (GC-QTOF) for monounsaturated fatty acid oxidation product quantification, or methylated, then treated with dimethyldisulfide (DMDS) and analyzed by GC-MS/MS to determine double-bond stereochemistry. *Cis* and *trans* isomers of monounsaturated fatty acid methyl esters react with DMDS stereospecifically to form threo and erythro adducts, which exhibit similar mass spectra, but are well-separated by gas chromatography, allowing unambiguous double-bond stereochemistry determination [59].

### 2.3. Derivatization

The derivatization method used was silylation allowing the replacement of the active hydrogen atom of carboxylic and alcoholic groups of acids and hydroxyacids by a trimethylsilyl group in one step. TLEs were silylated by dissolving them in 300 µL of a mixture of pyridine and BSTFA (N,O-Bis(triméthylsilyl)trifluoroacétamide; Supelco; 2:1, *v*/*v*) and heating to 50 °C (1 h). After evaporation to dryness under a stream of N_2_, the derivatized residue was dissolved in a mixture of hexane and BSTFA (to avoid desilylation) and analyzed by GC-MS/MS or GC-QTOF.

### 2.4. Determination of Double-Bond Stereochemistry

TLEs were dissolved in 2 mL of BF_3_/methanol (10%) (Sigma-Aldrich, St. Louis, MO, USA) and heated at 80 °C (1 h) in a screw-cap flask to obtain fatty acid methyl esters (FAMEs). After cooling, an excess of water was added and FAMEs were extracted three times with hexane, dried over anhydrous Na_2_SO_4_, filtered on Whatman cellulose filters (diameter 90 mm, porosity 11 µm), concentrated using rotary evaporation, and transferred to screw-cap flasks. After solvent removal (N_2_), 200 μL of DMDS (Sigma-Aldrich, St. Louis, MO, USA) and 50 μL of iodine solution (60 μg μL^−1^ in diethyl ether) were added. The mixtures were shaken and heated at 50 °C (48 h), excess iodine was removed by addition of 2 mL of a 5% Na_2_S_2_O_3_ solution, and lipids were extracted three times with 1 mL of hexane. The extracts were dried with anhydrous Na_2_SO_4_, filtered, and concentrated prior to analysis by GC–MS/MS.

### 2.5. 10S-DOX Degradation Estimate

Taking into account the production of equal amounts of 9-*trans* and 10-*trans* allylic hydroxyacids during the photooxidation and autoxidation of the Δ^9^ monounsaturated fatty acid [60,61] and their specific allylic rearrangement to 11-*trans* and 8-*trans* isomers, respectively [62], the contribution of the 10*S*-DOX degradation was obtained by difference between (10-*trans* + 8-*trans*) and (9-*trans* + 11-*trans*) oxidation products of palmitoleic acid [63].

### 2.6. Gas Chromatography/Tandem Mass Spectrometry

GC-MS and GC-MS/MS analyses were performed using an Agilent 7890A/7000A tandem quadrupole gas chromatograph system (Agilent Technologies, Parc Technopolis—ZA Courtaboeuf, Les Ulis, France). A cross-linked 5% phenyl-methylpolysiloxane (Agilent; HP-5MS, Agilent Technologies, Parc Technopolis—ZA Courtaboeuf, Les Ulis, France) (30 m × 0.25 mm, 0.25 μm film thickness) capillary column was used. Analyses were performed with an injector operating in pulsed splitless mode set at 270 °C and the oven temperature was programmed from 70 °C to 130 °C at 20 °C min^−1^, then to 250 °C at 5 °C min^-1^ and then to 300 °C at 3 °C min^−1^. The pressure of the carrier gas (He) was maintained at 0.69 × 10^5^ Pa until the end of the temperature program and then programmed from 0.69 × 10^5^ Pa to 1.49 × 10^5^ Pa at 0.04 × 10^5^ Pa min^−1^. The mass spectrometric conditions were: electron energy, 70 eV; source temperature, 230 °C; quadrupole 1 temperature, 150 °C; quadrupole 2 temperature, 150 °C; collision gas (N_2_) flow, 1.5 mL min^−1^; quench gas (He) flow, 2.25 mL min^−1^; mass range, 50–700 Daltons; cycle time, 313 ms. Quantification was carried out with external standards in multiple reaction monitoring (MRM) mode. Precursor ions were selected from the more intense ions (and specific fragmentations) observed in electron ionization (EI) mass spectra.

### 2.7. Gas Chromatography-EI Quadrupole Time of Flight Mass Spectrometry

Accurate mass measurements were carried out in full scan mode using an Agilent 7890B/7200 GC-QTOF System (Agilent Technologies, Parc Technopolis—ZA Courtaboeuf, Les Ulis, France). A cross-linked 5% phenyl-methylpolysiloxane (Agilent; HP-5MS ultra inert) (30 m × 0.25 mm, 0.25 μm film thickness) capillary column was used. Analyses were performed with an injector operating in pulsed splitless mode set at 270 °C and the oven temperature was programmed from 70 °C to 130 °C at 20 °C min^−1^ and then to 300 °C at 5 °C min^−1^. The pressure of the carrier gas (He) was maintained at 0.69 × 10^5^ Pa until the end of the temperature program. Instrument temperatures were 300 °C for the transfer line and 230 °C for the ion source. Nitrogen (1.5 mL min^−1^) was used as collision gas. Accurate mass spectra were recorded across the range *m*/*z* 50–700 at 4 GHz with the collision gas opened. The QTOF-MS instrument provided a typical resolution ranging from 8009 to 12,252 from *m*/*z* 68.9955 to 501.9706. Perfluorotributylamine (PFTBA) was used for daily MS calibration. Compounds were identified by comparing their TOF mass spectra, accurate masses, and retention times with those of standards. Each compound was quantified by extraction of specific accurate fragment ions, peak integration, and determination of individual response factors using external standards and Mass Hunter software (Agilent Technologies, Parc Technopolis—ZA Courtaboeuf, Les Ulis, France).

### 2.8. Statistical Analysis

The variable investigated being non-parametric, Spearman correlations were performed to determine the correlation between the depth and the *trans/cis* ratio or 10*S*-DOX degradation percentage. Mann–Whitney–Wilcoxon tests were performed to identify any significant differences in (i) *trans/cis* ratio and 10*S*-DOX degradation percentage between Baffin Bay and the Beaufort Sea and (ii) sterol:stanol ratio percentage between sitosterol and brassicasterol.

## 3. Results and Discussion

### 3.1. Contribution of Sympagic Material to Arctic Sediments

The main fatty acids detected in the TLEs of the different sediments examined were C_14:0_, C_16:0_, C_18:0_, C_16:1ω7_ (palmitoleic), C_18:1ω9_ (oleic), and C_18:1ω7_ (vaccenic) acids. These results are in close agreement with previous observations in surface sediments from the Beaufort Sea [64] and the Baffin Bay (Amiraux, unpublished data). Concentrations of palmitoleic acid (well-known to be the main fatty acid component of diatoms [65,66]) and vaccenic acid (typically of bacterial origin, [33,34]) were highly variable ranging from 0.2 to 1220 µg g^−1^ and 0.5 to 149 µg g^−1^, respectively (Supplementary Table S1). As previously observed [8,64], sediments of the Beaufort Shelf, which are under the influence of the Mackenzie River, had high contents of classical tracers of terrestrial higher plants (e.g., betulin, amyrins, dehydroabietic acid, and cutin components [67,68]).

**Figure 2:**
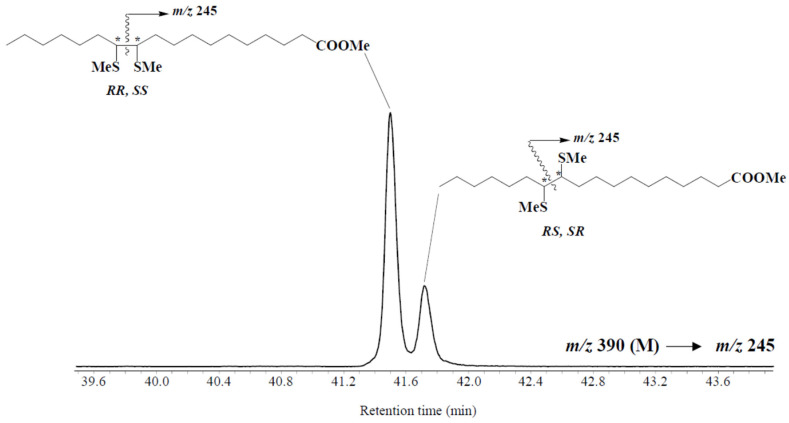
MRM chromatograms (*m/z* 390 → 245) of DMDS derivatives of vaccenic acid in superficial bottom sediment (0–1 cm) collected at St. 428. (*) asymmetric carbon atoms.

*Trans/cis* ratios of vaccenic acid were measured after DMDS treatment in the different surface sediment samples investigated (Figure 2). The results obtained (Figure 1, Table 1, and Supplementary Table S2) showed significantly higher values in the Beaufort Sea than in Baffin Bay (mean ± SE = 0.20 ± 0.03 and 0.06 ± 0.02 (g:g), respectively; W = 210, *p* < 0.01). The efficiency of sympagic–benthic coupling should logically be better in shallower zones. This assumption is well supported by the good anticorrelation observed between *trans/cis* ratio and depth of the different stations (Spearman’s rho = −0.67, *p* < 0.01).

A strong contribution sympagic material to the sediments of the Beaufort Sea is well supported by the high ratios previously measured in surface sediments of other stations in this zone (0.65 ± 0.15 g:g, *n* = 6) [39]. It was previously observed that bacterial osmotic stress resulting from hypersaline conditions in brine channels occurred only during the early stages of ice melting and not at the end of the melting season [7,14]. The sympagic material reaching the seafloor of the Beaufort Sea thus seems to be discharged from sea ice at the beginning of melting. The high *trans/cis* ratios observed (Figure 1; Table 1) ruled out a significant contribution of sympagic algae released in the water column at the advanced stages of ice melting and open water phytoplankton to the sediments. A strong contribution of such material (exhibiting very low and no CTI activity, respectively [14,69]) to the sediment should strongly lower the *trans/cis* ratios (by adding bacteria unstressed by salinity with *trans/cis* ratios < 0.1). This assumption is firmly supported by the results of Morata et al. [70], who observed that inputs of sympagic algae to the sediment started to increase from January/February in the Beaufort Sea, while sympagic algal bloom occurred between mid-March and late May [71]. Juul-Pedersen et al. [72] also observed a sinking export of sympagic algal material in the same zone during winter and early spring (i.e., well before the onset of spring melt). The amount of diatoms exported to the seafloor strongly depends on a match or mismatch between algal production and zooplankton grazing [73]. It is generally considered that copepods do not feed at chlorophyll concentrations < 1 µg L^−1^ [74].

The very low chlorophyll concentrations measured in POM between February and March in the Beaufort Sea (0.002–0.003 µg L^−1^ [70]) should thus not favor zooplankton grazing on sympagic algae during this period. We note that an increase in zooplankton fecal pellet production was observed during this same period [75]; however, those authors attributed it to the grazing of non-pigmented sources of food, such as microzooplankton.

Chlorophyll concentration increases strongly in surface waters in late spring and summer at the end of ice melting and during open water diatom bloom (up to 5 µg L^−1^ [76]) favoring grazing of sympagic [77] and pelagic algae by copepods. Coprophagy can then reduce losses by sinking of the resulting fecal pellets at only a few percent of the pellet production rate [78]. Bacterial decomposition by non-stressed internal bacteria is another mechanism that could prevent pellets sinking to the seafloor [79,80]. Forest et al. [81] previously estimated that 97% of the primary-produced C was grazed or degraded in the water column of the Amundsen Gulf. The remaining 3% reaching the seafloor thus seem mainly composed of sympagic algae associated with bacteria stressed by salinity and released during the early stages of ice melting. This view is well supported by earlier observations of Morata and Renaud [82]. These authors analyzed sedimentary pigments in the Beaufort Sea and concluded that in the spring, sympagic-algal production largely influenced organic matter inputs to the benthos, while in the summer grazing was responsible for inputs of degraded material.

Whereas a high CTI activity was previously observed in sea ice and sinking particles during the early stages of ice melting and in sediments from a landfast ice station located at Qikiqtarjuaq near Broughton Island (western Baffin Bay, [7]), *trans/cis* ratios were found to be particularly weak in middle and eastern Baffin Bay (Figure 1). Interestingly, Yunda-Guarin et al. [8] found more sea ice-derived particulate organic carbon in surface sediments in the western side than in the eastern side of Baffin Bay. These authors attributed this difference to the timing of sea ice retreat. Ice cover is greater in the western part of Baffin Bay than in its eastern side, which is influenced by warmer waters from the western streams of Greenland [83]. Moreover, recently calculated ecological network analysis indices reveal that the complex eastern Baffin Bay food web favors the classical grazing chain, while the shorter western food web induces a higher carbon export [84]. Based on these results, the contrasting high *trans/cis* ratio observed in sediments from Qikiqtarjuaq (Figure 1) may be due to a large contribution of sympagic material containing bacteria stressed by salinity. This hypothesis is supported by the similarity of *trans/cis* ratios previously observed at this station in sinking particles and in surface sediments [7]. The very low ratios observed in the other samples from eastern Baffin Bay (Figure 1) are in close agreement with the higher contributions of pelagic carbon observed by Yunda-Guarin et al. [8] in these zones.

**Figure 3:**
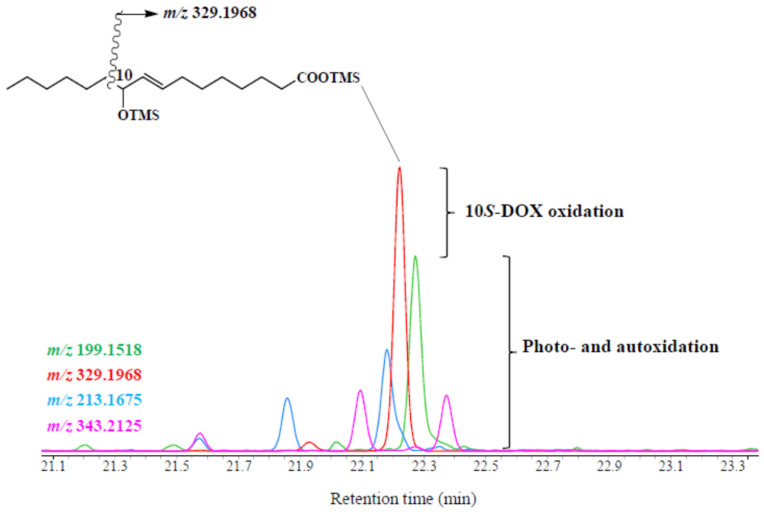
Partial ion chromatograms (at *m/z* 199.1518, 329.1968, 213.1675, and 343.2125) showing the presence of biotic and abiotic palmitoleic oxidation product trimethylsilyl derivatives (including the 10*S*-DOX degradation product, 10*S*-hydroxyhexadec-8(*trans*)-enoic acid) in superficial bottom sediment (0–1 cm) collected at Qikiqtarjuaq.

10*S*-Hydroxyhexadec-8(*trans*)-enoic acid arising from NaBH_4_ reduction in the corresponding hydroperoxide was detected in most of the sediments investigated (Figure 1 and Figure 3; Supplementary Table S2). The percentages of this hydroxyacid (relative to the parent palmitoleic acid and its degradation products) observed in Baffin Bay and the Beaufort Sea were not significatively different (mean ± SE = 15.20 ± 2.80 and 13.64 ± 1.80%, respectively; W = 130.5, *p* = 0.69). The presence of this compound is indicative of bacterial 10*S*-DOX oxidation of palmitoleic acid [85]. This enzymatic activity seems to be a detoxification strategy enabling sympagic bacteria to survive the production of deleterious free palmitoleic acid [33,86] by sympagic algae [14]. We note that despite this detoxification strategy a high mortality of attached bacteria could previously be observed in sea ice during the sympagic bloom [14]. It was recently observed that 10*S*-DOX could also be employed by some pelagic bacteria to detoxify free palmitoleic acid released by wounded diatoms in the presence of copepods [38]. The lack of correlation observed between the percentage of 10*S*-hydroxyhexadec-8(*trans*)-enoic acid and depth (Spearman’s rho = −0.22, *p* = 0.25) suggests a mixed contribution of sympagic and pelagic bacteria to the sedimentary 10*S*-DOX signal. Although 10*S*-hydroxyhexadec-8(*trans*)-enoic acid is thus not sufficiently specific for use as a marker of the contribution of sympagic material to Arctic sediments, it is indicative of the presence of bacteria whose membranes are altered by FFAs. The bacterial communities present in Arctic sediments are thus in part non-growing (i.e., stressed by salinity in brine channels of ice) or dead (i.e., stressed by FFAs in ice or in the water column).

It is generally considered that the deposited algal aggregates are overgrown within a few weeks to months by specific bacterial groups of the surrounding sediment [87], which are likely better adapted to deep-sea environment than surface-derived bacteria [88]. In the Arctic, these benthic bacteria are dominated by members of the Roseobacter clade (i.e., the genera *Neptunomonas*, *Arcobacter,* and *Sedimentitalea* [87]), which all contain large proportions of *cis*-vaccenic acid [89,90,91]. The lack of dilution of *trans/cis* ratios of this acid observed in surface sediments from the Beaufort Sea and northern and western Baffin Bay (Figure 1; Table 1) is thus very surprising. In these zones, sympagic-derived aggregates reaching the seafloor do not appear to be intensively colonized by benthic bacteria. This absence of colonization may be attributed to the presence of bactericidal FFAs (notably palmitoleic acid) in these aggregates attested by the 10*S*-DOX activity observed in most of them (Figure 1; Supplementary Table S2).

### 3.2. Impact of Stress State of Bacteria on the Degradation of Sympagic Material in Sediments

It is generally considered that the lifetime of organic compounds in marine sediments depends on environmental conditions such as bioturbation, physical mixing, and the presence or absence of oxygen and other electron acceptors [92]. The benthic fauna inhabiting the seafloor is known to: (i) modify the vertical zonation of respiration reactions, (ii) control microbial community assembly, (iii) change the distribution of organic matter (OM), and (iv) influence rates of microbial OM remineralization in surface sediments [93]. Preservation/degradation of OM also depends on its sources [94]; terrestrial vascular debris generally contain greater proportions of refractory organic carbon than algal material, owing to their geochemical composition [95]. The non-growing or dead state of bacteria associated with sympagic algae should strongly impact the degradation conditions of this material in Arctic sediments. Since the conversion of Δ^5^-stenol to stanol is generally considered as indicating an intense bacterial degradation [96,97], we compared the stanol/Δ^5^-stenol ratios of 24-methylcholesta-5,22-dien-3β-ol (brassicasterol) (common sterol of sympagic diatoms; [98]) and 24-ethylcholest-5-en-3β-ol (sitosterol) (sterol present in some diatoms but also widely distributed in terrestrial vascular plants [99]) in sediment samples from the Beaufort Sea where the highest bacterial stresses were observed (Table 1). We note that in these sediments sitosterol is known to result mainly from terrigenous material [64,100]. The stanol/Δ^5^-stenol ratios obtained for the two sterols were significantly different (mean ± SE = 18.2 ± 1.9% and 52.8 ± 5.9%, for brassicasterol and sitosterol respectively; W = 36, *p* < 0.01, Table 2). In the case of brassicasterol, the values observed were close to those found in healthy phototrophic organisms (5–20% [101,102]), attesting to the good biotic preservation of the algal material. By contrast, the measured sitostanol/sitosterol ratio reflects a strong biodegradation of this terrigenous sterol and supports the results of Gõni et al. [103], who estimated that 65% of the old and fossil carbon is respired or buried in the Mackenzie delta. On entering the sea, terrestrial organic matter thus seems to undergo strong bacterial remineralization. This could be attributed to the involvement of “priming effects” (enhanced remineralization of terrestrial OM in the presence of fresh substrates from pelagic algal sources; [104,105,106]). By contrast, aggregated sympagic material sinking quickly through the water column with bacteria of poor health status appears well-preserved in surface sediments.

**Figure 4:**
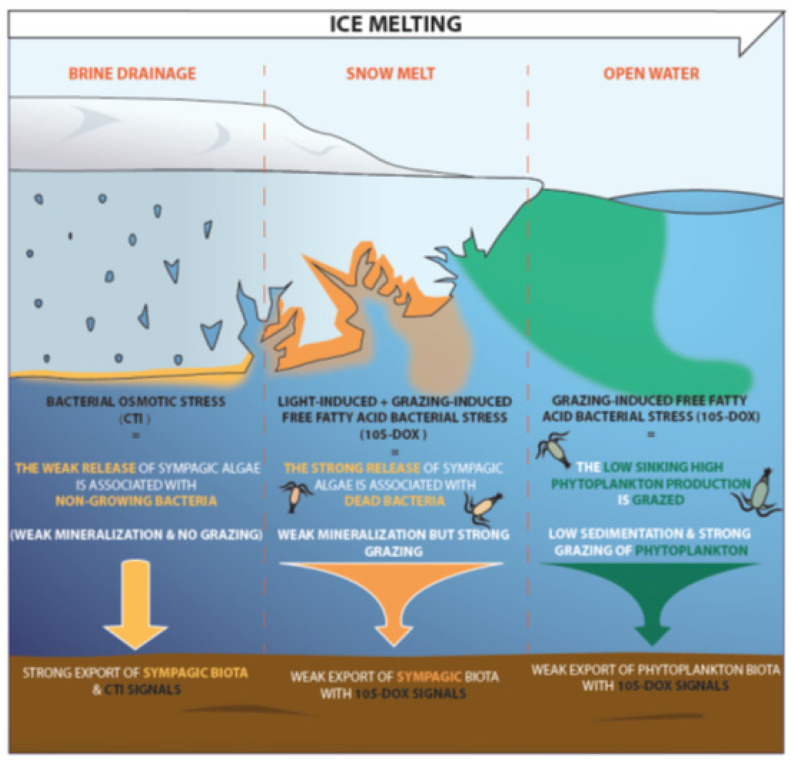
Conceptual scheme summarizing bacterial stress and its consequences on sympagic algae export in the Beaufort Sea and northern and western Baffin Bay during the sea ice melting stages: brine drainage, snowmelt, and open water.

Under warmer conditions, the Arctic carbon cycle, which is mostly driven by the primary production (sympagic and pelagic algae) and the riverine inputs (including permafrost [107]), will be impacted. Although the contributions of these sources to the exported carbon have recently been estimated [2,108,109,110], their burial efficiency, impacting Arctic CO_2_ storage remains uncertain. Here, we monitored their biotic behavior with specific lipid tracers in several surface sediment samples collected in the Canadian Arctic. We show that in the Beaufort Sea and northern and western Baffin Bay, unlike phytoplankton and permafrost, sympagic algae organic matter released during the early stages of ice melt by brine drainage contributes significantly to the exported and buried carbon, owing to: (i) the weak zooplanktonic grazing activity during this period, (ii) its strong aggregation enhancing rates of sinking to the seafloor, and (iii) its recalcitrance to demineralizing processes. This particular resistance to biodegradation results from the non-growing (inactive) and dead state of bacteria associated with this sympagic material (Figure 4).

We note that Koch et al. [111] previously estimated fluxes of sympagic diatoms in northern Bering and Chukchi Seas with HBI tracers and observed the highest fluxes in July (i.e., at the end and not in the early stages of ice melting). These observations contrasting with ours may be attributed to the particularly strong pelagic–benthic coupling of northern Bering and Chukchi Seas resulting from low grazing pressure due to temporal mismatch between zooplankton and sympagic algae production [112] and the shallowness of these zones [113]. However, an effect of the climate of specific years when the research was carried out cannot be totally excluded. Match or mismatch of zooplanktonic grazing with sympagic algae fluxes seems to be a true key factor controlling the export of sympagic material to the seafloor (Figure 4). In the future, it will be of interest to analyze several Arctic sediments to determine whether the conclusions of this work can be extended to other Arctic Zones. During these comparative studies, it will be necessary to use the same research protocol (i.e., the same tracers).

## Figures and Tables

**Table 1:** Percentage of 10*S*-DOX degradation of palmitoleic acid and *trans/cis* ratio of vaccenic acid observed in sediment samples collected in the Canadian Arctic during the ArcticNet, Malina, and GreenEdge expeditions.

	Beaufort Sea Mean ± SE (Range)	Baffin Bay Mean ± SE (Range)	Overall Mean ± SE (Range)
*Trans/cis* ratio (g:g)	0.20 ± 0.03 ^a^ (0.46–0.07)	0.06 ± 0.02 ^b^ (0.23–0.01)	0.14 ± 0.02 ^c^ (0.46–0.01)
10*S*-DOX (%) ^d^	14.34 ± 1.83 (29.00–2.20)	13.56 ± 2.91 (29.70–0)	14.01 ± 1.61 (29.70–0.00)

^a^ *n* = 17. ^b^ *n* = 13. ^c^ *n* = 30. ^d^ Relative to the residual palmitoleic acid and its abiotic degradation products.

**Table 2:** Stanol/stenol ratio of sitosterol and brassicasterol in sediment samples collected in the Canadian Arctic.

	Sitosterol Mean ± SE (Range)	Brassicasterol Mean ± SE (Range)
Stanol/stenol ratio (%)	52.8 ± 5.9 ^a^ (84.6–14.3)	18.2 ± 1.9 ^a^ (35.8–5.5)

^a^ *n* = 17.

## Data Availability

Not applicable.

## Supplementary Materials

The following are available online at https://www.mdpi.com/article/10.3390/microorganisms9122626/s1, Table S1: Depth, coordinates, and concentrations (µg g^−1^) of the main lipids of interest in the different surface sediments investigated, Table S2: Depth, coordinates, *trans*-vaccenic acid/*cis*-vaccenic acid ratio, and percentage of 10*S*-DOX product of the different surface sediments investigated.
