# Supplementary material for: Use of Stress Signals of Their Attached Bacteria to Monitor Sympagic Algae Preservation in Canadian Arctic Sediments
Source: Microorganisms. 2021 Dec 20;9(12):2626. doi: 10.3390/microorganisms9122626 (PMC8704379; doi:10.3390/microorganisms9122626)
Supplement: Supplementary file 1 [file microorganisms-09-02626-s001.zip › microorganisms-1500104-supplementary.pdf]

Table S1
Depth, coordinates, and concentrations (μg g^{-1}) of the main lipids of interest[a] in the different surface sediments investigated.

Station	Depth (m)	Latitude (°N)	Longitude (°W)	Brassicasterol	24-Methylene Cholesterol	Sitosterol	Palmitoleic acid	Vaccenic acid[c]
719	1948	69.51	63.23	0.16	0.11	0.07	3.20	0.68
600	2106	70.51	62.01	1.09	0.62	0.89	1.82	0.74
605	2017	70.49	61.58	0.65	0.37	0.44	5.47	0.91
615	617	70.50	58.47	1.06	0.43	0.69	3.47	0.91
713	1892	69.50	61.58	0.09	0.06	0.08	1.93	2.93
707	1419	69.51	59.81	1.66	0.87	0.89	8.11	1.73
Qik	360	67.47	63.79	0.11	0.11	0.08	57.07	26.98
403	1672	68.03	60.40	0.36	0.28	0.27	0.30	0.18
409	1404	68.10	58.00	1.07	0.46	0.47	2.39	1.40
418	380	68.11	56.23	2.37	0.75	0.38	2.76	0.53
680	115	69.61	138.21	0.50	0.30	1.10	1220.85	149.06
110	385	71.70	126.48	3.00	1.50	6.20	30.70	80.49
3	811	74.48	79.75	0.12	0.03	0.07	6.87	1.89
4	347	74.27	91.77	0.28	0.45	0.37	30.36	1.76
NOW	703	77.84	74.68	0.02	tr	0.23	6.90	1.14
111	608	76.30	73.11	0.76	0.40	0.23	65.87	11.20
115	615	76.30	71.67	0.20	0.21	0.08	16.28	5.29
6	61	69.18	100.70	1.20	0.86	1.11	18.54	1.11
7	112	69.00	106.57	0.03	0.04	0.03	0.32	0.99
314	93	69.07	106.57	0.11	0.15	0.13	1.99	3.04
1000	362	70.60	120.92	0.02	0.01	0.02	5.88	1.49
405b	513	70.57	123	tr[b]	tr	0.14	3.37	1.13
408	168	71.32	127.65	tr	tr	0.01	2.62	1.72
12	219	69.92	122.98	0.04	0.06	0.09	1.65	1.70
1116	184	70.05	126.29	0.01	0.01	0.02	6.94	2.33
1122	15	70.48	127.59	0.01	0.01	0.10	13.55	1.00
434	7	70.17	133.59	0.70	0.33	0.49	426.67	34.89
1214	223	70.73	127.36	0.02	0.02	0.03	0.24	0.82
428	43	70.79	133.74	0.05	0.03	0.06	65.33	3.83
1800	352	72.20	127.82	0.16	0.16	0.17	4.36	6.06

[a] Unspecific lipids (such as saturated fatty acids, oleic acid and cholesterol) were not quantified.
[b] tr = traces (concentrations < 0.01 μg g^{-1}).
[c] Total (*cis* + *trans*)

Table S2

Trans-vaccenic acid/*cis*-vaccenic acid ratio and percentage of 10*S*-DOX product of the different surface sediments investigated.

Station	*Trans*-vaccenic acid/*cis*-vaccenic acid	10*S*-DOX (%)[a]
719	0.02	1.0
600	0.02	5.6
605	0.02	6.4
615	0.01	3.0
713	0.01	0.0
707	0.05	12.4
Qik	0.23	26.8
403	0.06	6.2
409	0.06	20.0
418	0.02	22.1
680	0.22	5.2
110	0.46	2.2
3	0.12	22.8
4	0.15	29.0
NOW	0.16	29.7
111	0.04	18.3
115	0.13	24.8
6	0.20	9.4
7	0.22	26.2
314	0.18	13.9
1000	0.11	15.0
405b	0.12	6.3
408	0.12	17.0
12	0.35	18.3
1116	0.24	17.5
1122	0.20	10.0
434	0.07	10.0
1214	0.12	6.6
428	0.36	14.0
1800	0.09	20.5

[a] Percentage of 10*S*-hydroxyhexadec-8(*trans*)-enoic acid relative to residuel palmitoleic acid and its abiotic oxidation products.
